# Supplementary material for: Lead Exposure Can Affect Early Childhood Development and Could Be Aggravated by Stunted Growth: Perspectives from Mexico
Source: Int J Environ Res Public Health. 2023 Mar 15;20(6):5174. doi: 10.3390/ijerph20065174 (PMC10049063; doi:10.3390/ijerph20065174)
Supplement: Supplementary file 1 [file ijerph-20-05174-s001.zip › ijerph-2239804-supplementary.pdf]

Table S1: Child, maternal, household, and family care characteristics by regions

	Total n sample = 1,394 N = 2,415,000 (%)	95% CI	Regions: North n sample = 256 N = 437,500 (15.3%)	95% CI	Regions: Center n sample = 437 N = 1,945,000 (32.9%)	95% CI	Regions: South n sample = 709 N = 1,945,000 (51.8%)	95% CI	p-value
Child characteristics									
**Mean Language z-score (SE)**	**-0.08 (0.07)**	**-0.15, -0.05**	**0.18 (0.15)**	**-0.13, 0.49**	**-0.06 (0.11)**	**-0.28, 0.15**	**-0.17 (0.09)**	**-0.35, 0.002**	**0.0001[d]**
Blood lead level									
Non-detectable (< 3.3 μg/dL)	803 (50.3)	39.5, 61.0	150 (63.6)	52.5, 73.4	224 (54.9)	46.1, 63.5	429 (43.4)	39.5, 61.4	0.15[e]
Detectable (≥ 3.3 μg/dL)	583 (49.7)	38.9, 60.5	90 (36.4)	26.5, 47.5	213 (45.0)	36.4, 53.9	280 (56.6)	38.9, 60.5	
Age (months)									
12 – 23	292 (29.1)	19.2, 41.6	47 (16.5)	11.2, 23.6	99 (26.2)	20.6, 32.7	146 (34.8)	18.4, 55.9	0.31[e]
24 – 35	350 (21.7)	16.4, 28.1	68 (29.3)	17.8, 44.2	100 (23.7)	15.8, 33.8	182 (18.2)	11.7, 27.4	
36 – 47	375 (28.1)	22.3, 34.8	67 (31.7)	21.8, 43.7	122 (25.5)	18.8, 33.6	186 (28.7)	19.3, 40.3	
48 – 59	369 (21.0)	15.8, 27.4	58 (22.5)	15.9, 30.9	116 (24.6)	18.6, 31.8	195 (18.3)	10.6, 29.8	
Sex									
Girl	657 (52.1)	44.3, 59.8	110 (41.6)	30.1, 54.2	206 (54.2)	47.3, 60.9	341 (53.9)	44.3, 59.8	0.34[e]
Boy	729 (47.9)	40.1, 55.7	130 (58.4)	45.7, 69.9	231 (45.8)	39.1, 52.7	368 (46.1)	40.1, 55.7	
Maternal characteristics									
**Maternal schooling (%)[a]**									
**Middle school or higher**	**870 (76.6)**	**69.7, 82.3**	**186 (90.5)**	**839, 94.6**	**299 (70.2)**	**53.5, 76.2**	**385 (76.5)**	**63.6, 85.8**	**0.03[e]**
**Elementary school**	**437 (23.4)**	**17.6, 30.3**	**43 (9.5)**	**5.4, 16.1**	**115 (29.8)**	**23.8, 36.5**	**279 (23.5)**	**14.2, 36.4**	
Household characteristics									
SES (%)[b]									
Lower	949 (62.2)	52.2, 71.3	128 (49.9)	35.5, 64.3	222 (50.6)	41.7, 59.4	599 (73.2)	56.4, 85.3	0.06[e]
Middle	339 (27.1)	20.0, 35.6	86 (36.5)	20.8, 55.7	161 (35.9)	28.0, 44.6	92 (18.8)	9.9, 32.8	
Higher	93 (10.7)	6.6, 16.8	26 (13.6)	7.1, 24.3	51 (13.6)	9.4, 19.2	16 (7.8)	2.7, 21.5	
Locality type									

Urban	210 (45.3)	33.9, 57.2	49 (47.9)	30.9, 65.3	67 (40.7)	33.0, 48.9	94 (47.4)	28.1, 67.5	0.72[e]
Rural	1176 (54.7)	42.8, 66.0	191 (52.1)	34.7, 69.0	370 (59.3)	51.1, 66.9	615 (52.6)	32.5, 71.8	
Family care characteristics									
Preschool education									
Yes	419 (21.9)	18.5, 25.7	55 (17.4)	10.4, 27.7	121 (23.2)	18.9, 28.2	243 (22.4)	17.1, 28.7	0.54[e]
No	967 (78.1)	74.3, 81.4	185 (82.6)	72.2, 89.6	316 (76.8)	71.8, 81.1	466 (77.6)	71.3, 82.8	
Percentage of children with learning support (%)									
Yes	896 (75.4)	68.6, 81.2	182 (83.7)	76.3, 89.1	273 (70.2)	63.4, 76.2	441 (76.3)	63.7, 85.5	0.19[e]
No	490 (24.6)	18.8, 31.4	58 (16.3)	10.9, 23.7	164 (29.8)	23.8, 36.6	268 (23.7)	14.5, 36.3	
Percentage of children with at least three books (%)									
≥ 3	226 (20.6)	15.1, 27.4	52 (25.8)	17.3, 36.6	85 (21.5)	14.6, 30.4	89 (18.5)	10.6, 30.5	0.57[e]
< 3	1160 (79.4)	72.6, 85.9	188 (74.2)	53.4, 82.7	352 (78.5)	69.5, 85.4	620 (81.5)	69.5, 89.4	
Percentage of children with two or more toy types (%)									
≥ 2	1338 (96.6)	93.5, 98.2	237 (98.4)	97.7, 98.9	426 (96.0)	86.8, 98.9	675 (96.4)	92.2, 98.4	0.56[e]
< 2	48 (3.4)	1.8, 6.4	3 (1.6)	1.1, 2.3	11 (3.9)	1.1, 13.2	34 (3.6)	1.6, 7.8	
Children disciplined with violent methods (%)									
Yes	766 (58.1)	48.6, 67.1	141 (57.1)	41.4, 71.4	243 (50.1)	43.2, 56.9	382 (63.5)	47.3, 77.1	0.25[e]
No	620 (41.9)	32.9, 51.4	99 (42.9)	28.6, 58.5	194 (49.9)	43.0, 56.7	327 (36.5)	22.9, 52.6	

[a] Missing value = 83
[b] Missing value = 6
[c] Missing value = 8
[d] T-test
[e] ANOVA

Table S2. Association between blood lead levels (three categories) and language z-score stratified by stunting condition

	**Not stratified analysis**		**Stratified analysis**			
	Complete sample<br>n sample = 1413<br>N = 2,415,000		Stunted children<br>n sample = 256<br>N = 437,500		Not stunted children<br>n sample = 1138<br>N = 1,945,000	
	β	95% IC	β	95% IC	β	95% IC
Child characteristics						
**Blood lead level**						
Lower than 3.3 µg/dL (n= 816)						
**3.3 – 5 µg/dL (n= 372)**	-0.16	-0.39, 0.05	**-0.67**	**-1.02, -0.32**	-0.17	-0.43, 0.07
Higher than 5 µg/dL (n= 225)	-0.08	-0.35, 0.17	-0.05	-0.32, 0.20	-0.12	-0.38, 0.13
Stunting condition[a]						
Stunting						
Not stunting	-0.19	-0.44, 0.05				
**Sex**						
**Girls**						
**Boys**	-0.08	-0.30, 0.13	**-0.29**	**-0.54, -0.05**	0.10	-0.12, 0.36
Maternal characteristics						
Maternal schooling[b]						
Middle school or higher						
Elementary	-0.04	-0.25, 0.16	-0.02	-0.36, 0.31	-0.05	-- 0.29; 0.18
Household characteristics						
SES[c]						
Lower						
Middle	-0.07	-0.47, 0.33	-0.41	-0.96, 0.14	0.03	-0.27, 0.34
Higher	-0.13	-0.44, 0.18	-0.53	-1.01, -0.05	-0.09	-0.42, 0.23
**Locality type**						
**Urban**						
**Rural**	-0.18	-0.41, 0.05	**-0.94**	**-1.22, -0.66**	-0.06	- 0.31; 0.17
Country region						
North						
Center	-0.15	-0.50, 0.19	-0.03	-0.61, 0.54	-0.18	-0.64, 0.28
South	-0.30	-0.65, 0.05	-0.25	-0.69, 0.19	-0.33	-0.82, 0.14
Family care characteristics						

Preschool education						
Yes						
No	-0.10	-0.29, 0.07	-0.02	-0.27, 0.32	-0.13	-0.34; 0.07
**Percentage of children with learning support**						
**Yes**						
**No**	-0.18	-0.37, 0.01	**-0.37**	**-0.67, -0.06**	-0.22	-0.45, 0.0009
**Percentage of children with at least three books**						
**≥ 3**						
**< 3**	**-0.32**	**-0.59, -0.04**	**-0.69**	**-1.31, -0.07**	-0.26	-0.56. 0.02
Percentage of children with two or more toys						
≥ 2						
< 2	-0.12	-0.72, 0.48	-0.23	-0.83, 0.37	-0.05	- 0.72, 0.62
Children disciplined with violent methods						
Yes						
No	-0.09	-0.29, 0.07	0.17	-0.08, 0.42	-0.16	-0.37, 0.04

[a] Stunting condition: not stunting (> -2 standard deviation height for age); stunting (<= -2 standard deviation height for age)
[b] Missing value = 83
[c] Missing value = 6

Table S3. Association between blood lead levels (three categories) and not being developmentally on-track according to ECDI

	n sample = 753<br>N = 1,300,000		
	OR	95% IC	p-value
Child characteristics			
**Blood lead level**			
Lower than 3.3 µg/dL (n=431)	1.00		
3.3 – 5 µg/dL (n=205)	0.57	0.28, 1.14	0.112
**Higher than 5 µg/dL (n=124)**	**2.32**	**0.99, 5.40**	**0.050**
Stunting condition			
Stunting	1.00		
Not stunting	0.78	0.33, 1.85	0.579
**Age (months)**	**0.92**	**0.87, 0.97**	**0.006**
Sex			
Girls	1.00		
Boys	0.99	0.53, 1.85	0.998
Maternal characteristics			
Maternal schooling[a]			
Middle school or higher	1.00		
Elementary	0.83	0.40, 1.73	0.629
Household characteristics			
**SES[b]**			
**Lower**	1.00		
**Middle**	**3.22**	**1.18, 5.76**	**<0.001**
**Higher**	**8.98**	**4.96, 16.2**	**<0.001**
Locality type			
Urban	1.00		
Rural	0.72	0.36, 1.42	0.347
**Country region**			
**North**	1.00		
**Center**	**2.78**	**1.05, 7.38**	**0.039**
**South**	**3.30**	**1.22, 8.90**	**0.018**
Family care characteristics			
Preschool education			
Yes	1.00		
No	1.08	0.50, 2.31	0.842
Percentage of children with learning support			
Yes	1.00		
No	0.93	0.41, 2.08	0.861
Percentage of children with at least three books			
≥ 3	1.00		
< 3	0.54	0.27, 1.07	0.080
Percentage of children with two or more toys			
≥ 2	1.00		
< 2	1.51	0.30, 7.57	0.613
**Children disciplined with violent methods**			
**Yes**	1.00		
**No**	**0.16**	**0.08, 0.31**	**<0.001**

ECDI: Early Childhood Development Index
Not on-track vs on-track
[a] Missing value = 55
[b] Missing value = 6
